# Effect of intermittent preventive treatment during pregnancy with sulfadoxine-pyrimethamine on maternal gestational weight gain in low-income and middle-income countries: a systematic review and individual participant data meta-analysis of randomised clinical trials

**DOI:** 10.1016/j.eclinm.2025.103279

**Published:** 2025-06-02

**Authors:** Enju Liu, Uttara Partap, Sachin Shinde, Dongqing Wang, Janaína Calu Costa, Ilana R. Cliffer, Molin Wang, Sudeer Kumar Nookala, Vishak Subramoney, Brittany Briggs, Davidson H. Hamer, Hellen Akurut, Alemayehu Argaw, Ulla Ashorn, Jobiba Chinkhumba, Meghna Desai, Titus H. Divala, Alison M. Elliott, Julie R. Gutman, Alain Hien, Lieven Huybregts, Richard Kajubi, Abel Kakuru, Simon Kariuki, Carl Lachat, Miriam K. Laufer, Mari Luntamo, Kenneth Maleta, Don P. Mathanga, Teddy Ochieng, Maria Ome-Kaius, Noel Patson, Dominique Roberfroid, Stephen J. Rogerson, Laéticia Céline Toe, Holger W. Unger, Emily L. Webb, Wafaie W. Fawzi

**Affiliations:** a Institutional Centers for Clinical and Translational Research, Boston Children's Hospital, Boston, MA, USA; b Division of Gastroenterology, Hepatology and Nutrition, Boston Children's Hospital, Harvard Medical School, Boston, MA, USA; c Department of Global Health and Population, Harvard T.H. Chan School of Public Health, Harvard University, Boston, MA, USA; d Department of Global and Community Health, College of Public Health, George Mason University, Fairfax, VA, USA; e Department of Epidemiology, Harvard T.H. Chan School of Public Health, Harvard University, Boston, MA, USA; f Department of Biostatistics, Harvard T.H. Chan School of Public Health, Harvard University, Boston, MA, USA; g Cytel Inc., India on behalf of Gates Foundation, Seattle, WA, USA; h DVPL Tech, Dubai, United Arab Emirates; i Certara USA, Inc. on behalf of Gates Foundation, Seattle, WA, USA; j Department of Global Health, Boston University School of Public Health, Boston, MA, USA; k Section of Infectious Diseases, Boston University Chobanian & Avedisian School of Medicine, Boston, MA, USA; l Center on Emerging Infectious Diseases, Boston University, Boston, MA, USA; m MRC/UVRI and LSHTM Uganda Research Unit, Entebbe, Uganda; n Department of Food Technology, Safety and Health, Ghent University, Coupure Links 653, 9000, Gent, Belgium; o Tampere University, Faculty of Medicine and Health Sciences, Arvo Ylpön katu 34, FIN-33014 Tampereen Yliopisto, Finland; p Malaria Alert Center, The Kamuzu University of Health Sciences (KUHeS), Blantyre, Malawi; q Malaria Branch, National Center for Emerging and Zoonotic Infectious Disease, Centers for Disease Control and Prevention, Atlanta, GA, USA; r Blantyre Malaria Project, Kamuzu University of Health Sciences, Malawi; s London School of Hygiene and Tropical Medicine, Keppel St, London, WC1E 7HT, UK; t Nazi Boni University, Bobo-Dioulasso, Burkina Faso; u Nutrition, Diets, and Health Unit – International Food Policy Research Institute, Washington DC, USA; v Infectious Diseases Research Collaboration Kampala, Uganda; w Kenya Medical Research Institute (KEMRI) Centre for Global Health Research, Kisumu, Kenya; x Center for Vaccine Development and Global Health, University of Maryland School of Medicine, USA; y School of Public Health and Family Medicine, University of Malawi, College of Medicine, 1 Mahatma Gandhi Road, Private Bag 360, Blantyre 3, Blantyre, Malawi; z Papua New Guinea Institute of Medical Research, Goroka, Papua New Guinea; aa Namur University, Department of Medicine, Namur, Belgium; ab Department of Infectious Diseases, University of Melbourne, The Doherty Institute, Melbourne, VIC, 3000, Australia; ac Nutrition and Metabolic Diseases Unit, Health Sciences Research Institute (IRSS), Bobo-Dioulasso, Burkina Faso; ad Global and Tropical Health Division, Menzies School of Health Research, Charles Darwin University, PO Box 41096, Casuarina, NT, 0811, Australia; ae Department of Nutrition, Harvard T.H. Chan School of Public Health, Harvard University, Boston, MA, USA

**Keywords:** Intermittent preventive treatment during pregnancy, Gestational weight gain, Randomised controlled trials, Meta-analysis, Low- and middle-income countries

## Abstract

**Background:**

Studies have consistently demonstrated beneficial effects of intermittent preventive treatment during pregnancy (IPTp) with sulfadoxine-pyrimethamine (SP) on reducing malaria infection and improving birth outcomes among pregnant women in endemic areas. However, data on its impact on maternal gestational weight gain (GWG) are very limited. We aimed to conduct a two-stage meta-analysis of individual participant data to examine the effect of IPT with SP on GWG compared to other antimalarial regimens.

**Methods:**

In this systematic review and individual participant data meta-analysis, we conducted electronic literature searches of PubMed, Embase, Web of Science, and the Cochrane Library to identify eligible RCTs among pregnant women. We did not apply any language or publication date restrictions in the search. The initial search was conducted on August 4th, 2021, and updated on February 15th, 2025. The study-level inclusion criteria were as follow: 1) the studies must be randomised controlled trials (RCTs), which could be individually randomised, cluster randomised, or a combination of both; 2) study participants were pregnant at enrollment or enrolled before pregnancy and followed up in pregnancy; 3) studies were conducted in a low-income, lower-middle-income, or upper-middle-income economy defined by the World Bank country classification for the 2021 fiscal year; 4) antimalaria and/or antibiotic interventions were provided during pregnancy; and 5) the intervention was provided alone or in combination with a co-intervention that was similar across arms. Since we focused on the intervention's effect on GWG in generally healthy pregnant women, we applied the following study-level exclusion criteria: 1) studies without any measures of maternal weight during pregnancy; and 2) studies conducted exclusively among women with pre-existing health conditions, such as anemia, human immunodeficiency virus (HIV) infection, or diabetes. Within each eligible trial, we further applied individual-level criteria to identify eligible individual participants, including 1) singleton pregnancies, 2) at least one weight measurement in the second or third trimesters, 3) known gestational ages at the time of weight measurements, and 4) availability of maternal height measure. Risk of bias for each trial was assessed using the Cochrane risk-of-bias tool, version 2 (RoB 2). GWG percent adequacy (%) and total weight gain (gram) at delivery were calculated according to the Institute of Medicine 2009 guidelines. Linear regression models were used to estimate mean difference (MD) and 95% confidence intervals (CIs) in GWG percent adequacy and total weight gain across intervention arms. Results from individual trials were pooled using fixed-effects inverse-variance meta-analysis models. This study is registered with PROSPERO, CRD42023428794.

**Findings:**

A total of 97 trials were identified in the search and sough for IPD, of them eight trials including 8550 pregnant women were included in the current analysis. Women who received IPTp with only 2 doses of SP had a greater GWG percent adequacy (MD: 5.61%; 95% CI: 2.61%, 8.60%; P = 0.0002; I^2^ = 84.26%), and total GWG in grams at delivery (MD: 702; 95% CI: 321, 1083; P = 0.0003; I^2^ = 83.78%) than those who received weekly chloroquine as prophylaxis. No significant differences in GWG percent adequacy (MD: −0.53%; 95% CI: −2.89%%, 1.83%; P = 0.66; I^2^ = 0.00%) or GWG grams (MD: −80; 95% CI: −380, 221; P = 0.60; I^2^ = 0.00%) were found between IPTp with 2-dose SP and monthly IPTp-SP (3-dose or more). Compared to women who received monthly IPTp-SP, those who received monthly IPTp with dihydroartemisinin-piperaquine (IPTp-DHA + PPQ) had a lower GWG percent adequacy (MD: −5.56%; 95% CI: −8.22%, −2.90%; P < 0.0001; I^2^ = 13.47%) and total GWG in grams (MD: −723; 95% CI: −1037, −410; P < 0.0001; I^2^ = 46.29%). Adding azithromycin to an antimalarial regimen was associated with a greater GWG percent adequacy (MD: 2.75%; 95% CI: 0.46%, 5.05%; P = 0.19; I^2^ = 0.00%) and total GWG in gram at delivery (MD: 485; 95% CI: 210, 760; P = 0.0005; I^2^ = 75.66%).

**Interpretation:**

Our findings suggest that monthly IPTp-SP has superior effect on GWG compared to weekly chloroquine or IPTp-DHA + PPQ in malaria-endemic areas. The result provides further evidence indicating that IPTp-SP improves maternal weight gain, an important determinant of fetal growth beyond its antimalarial effects. Due to the limited number of trials with weight and height measures available for the IPD meta-analysis we were likely underpowered to detect any significant difference between 2-dose SP and monthly IPTp-SP. More efforts are warranted to examine the potential beneficial effect of adding azithromycin or DHA + PPQ to the standard antimalarial regimens.

**Funding:**

Gates Foundation.

**Research in context**

**Evidence before this study**

Limited research has examined the impact of preventive antimalarial treatments on maternal gestational weight gain (GWG). In a preliminary search of PubMed, Embase, Web of Science, and the Cochrane Library, using search terms related to low- and middle-income countries (LMICs), pregnancy, trials, and anti-infection agents, conducted on August 4th, 2021, with no restrictions on language or publication date, we found that previous meta-analyses in the field primarily focused on comparing various antimalarial regimens concerning their effects on incidental malaria, birthweight, or preterm birth outcomes. We did not identify any meta-analysis that have evaluated the effect of these treatments on GWG. In this study, we conducted an individual participant data meta-analysis using existing data from eight randomised controlled trials conducted in LMICs to assess the effect of different preventive antimalarial treatment regimens on GWG.

**Added value of this study**

This study contributes valuable insights to the existing evidence by focusing specifically on the impact of preventive antimalarial treatments on maternal GWG. Our analysis revealed significant findings regarding different treatment regimens. Firstly, pregnant women who received the two-dose SP regimen experienced a greater increase in GWG compared to those on a weekly chloroquine regimen. Additionally, women who received monthly IPTp-SP in combination with azithromycin exhibited significantly higher GWG than those who received a similar antimalarial regimen without azithromycin. On the other hand, monthly IPTp with dihydroartemisinin piperaquine (DHA + PPQ) was associated with significantly lower GWG compared to monthly IPTp-SP. This meta-analysis is the first of its kind to examine the effects of antimalarial IPTp on GWG using individual participant data obtained from randomised controlled trials in low- and middle-income countries.

**Implications of all the available evidence**

Our findings provide further evidence supporting the World Health Organization's recommendation that pregnant women in malaria-endemic areas should receive monthly IPTp-SP starting in the second trimester. Considering the widespread adoption of IPTp-SP and the increasing SP resistance, more efforts are needed to identify alternative medications or combination therapy to reduce malaria infection and promote optimal GWG and birth outcomes in populations where resistance to SP by the parasite is prevalent. We were limited by the number of trials with weight and height measures available for the current analysis, and likely underpower to detect any significant difference between 2-dose SP and monthly IPTp-SP.

## Introduction

Infection during pregnancy poses a substantial risk to the mother, the fetus and newborn infant. Pregnant women in many low- and middle-income countries (LMICs) are susceptible to a variety of bacterial, viral and parasitic infections including malaria, a parasitic infection transmitted by mosquitoes. In 2022, it was estimated that 12.7 million (36%) of the 35.4 million pregnancies in 33 moderate and high transmission Africa countries were exposed to malaria infection.^1^ Malaria infection during pregnancy is associated with an increased risk of anemia, maternal death, fetal loss, stillbirth and low birthweight.^2^, ^3^, ^4^ There are five main types of human malaria, each caused by a different species of *Plasmodium (P.)*. *P. falciparum* is the most widespread and dangerous form of malaria, and it is commonly seen in sub-Saharan Africa but also spreads in Southeast Asia and other regions; *P. vivax* is prevalent in Asian and Latin America and some parts of Africa; *P. malariae* is found worldwide but relatively uncommon; *P. ovale* is primary in West Africa; and *P. knowlesi* is mainly prevalent in Southeast Asia, like Borneo Island, Malaysia. While placental malaria caused by *P. falciparum* is an established pathway associated with poor pregnancy outcomes, the infection could also lead to adverse outcomes among pregnant women in LMICs by aggravating underlying malnutrition thereby preventing sufficient maternal gestational weight gain (GWG).^5^

To prevent adverse pregnancy consequences of malaria infection, the World Health Organization (WHO) has recommended that pregnant women in malaria-endemic areas receive IPTp-SP starting in the second trimester as early as possible, with repeated doses and at least 1 month apart.^6^ To date, 35 countries in the WHO African Region have adopted IPTp-SP to reduce the burden of malaria during pregnancy. Due to widespread resistance to SP by *P. falciparum*, IPTp with dihydroartemisinin piperaquine (DHA + PPQ) has been evaluated as an alternative in sub-Saharan Africa and has been found to have an antimalarial effect that is non-inferior to IPTp-SP.^7^, ^8^, ^9^ Furthermore, azithromycin, an antibiotic with antimalarial activity typically used to treat bacterial infection, has been explored in combination with SP or DHA + PPQ to reduce the risk of malaria and adverse pregnancy outcomes in these settings.^10^, ^11^, ^12^

Apart from its role in preventing and controlling malaria, antimalarial treatments during pregnancy may improve GWG by reducing placental malaria, increasing nutrient intake and absorption, thereby stimulating fetal growth. Results from recent research have indicated that SP probably influences birthweight and early infant growth via two distinct pathways, one is through its antimalarial activity, i.e. reducing placental malaria, the other is though non-malarial mechanisms.^13^^,^^14^ For example, SP has antibacterial activity which could reduce other infections during pregnancy; In addition, through impacting maternal gut microbiome SP might promote intestinal nutrient absorptions, thereby increasing nutrients available for maternal weight gain and fetus growth. Existing trials of antimalarial regimens have primarily investigated the effects on malarial infection and birth outcomes, very few studies have specifically examined their effect on GWG. In this individual participant data (IPD) meta-analysis, our objective was to assess the effect of different antimalarial preventive treatment regimens compared to the standard IPTp-SP on GWG. We achieved this by utilizing existing data from randomised controlled trials (RCTs) conducted in LMICs.

## Methods

### Search strategy and selection criteria

We conducted electronic literature searches using PubMed, Embase, Web of Science, and the Cochrane Library to identify eligible (randomised controlled trials) RCTs among pregnant women. The search strategy included terms for LMICs, pregnancy, trials, and anti-infection agents. We did not apply any language or publication date restrictions in the search, the details of search terms are presented in supplementary material. The search was conducted on August 4th, 2021 in English without language or date restrictions. We also reviewed the references of the included trials and previous systematic reviews to identify additional relevant studies. The search was updated on February 15th, 2025.

Two team members independently screened the titles and abstracts of the identified studies on Covidence, with any discrepancies resolved by discussion. After initial title and abstract screening, full text reviews were conducted for the remaining studies to confirm final eligibility. The study-level inclusion criteria were: 1) RCTs, which could be individually randomised, cluster randomised, or a mixture of individual and cluster randomization; 2) study participants were pregnant at enrollment or enrolled before pregnancy and followed up in pregnancy; 3) studies were conducted in a low-income, lower-middle-income, or upper-middle-income economy defined by the World Bank country classification for the 2021 fiscal year; 4) antimalaria and/or antibiotic interventions were provided during pregnancy; and 5) the intervention was provided alone or in combination with a co-intervention that was similar across arms. Exclusion criteria were: 1) studies without any measures of maternal weight during pregnancy; and 2) studies conducted exclusively among women with pre-existing health conditions, such as anemia, human immunodeficiency virus (HIV) infection, or diabetes.

We initiated collaboration with authors of eligible trials and requested the sharing of individual-level data. We worked with their respective institutions to establish suitable data-sharing agreements for those who agreed to participate. After obtaining data from each study, we ensured data completeness and reviewed relevant variables. Any data queries were addressed with individual principal investigators. To facilitate pooling of data across trials, data items were recoded into a common format and classifications of participant characteristics and disease/condition status were standardised. The process was supported by the Knowledge Integration team at Gates Foundation.

Most of the identified trials have been designed to examine the effect of antimalaria agents on maternal malaria infection and pregnancy and birth outcomes. For the current analysis focused on GWG, individual-level criteria were further applied to identify eligible individual participants, including 1) singleton pregnancies, 2) at least one weight measurement in the second or third trimesters, 3) known gestational ages at the time of weight measurements, and 4) availability of maternal height measure. After these exclusions, the balance across intervention arms with respect to baseline patient characteristics were checked for each trial separately (Supplementary Tables S1 and S2).

### Data analysis

We recorded intervention arms from each trial, then summarised and identified common or similar intervention arms across trials based on intervention medication and frequency of administration. We conducted meta-analyses for any comparisons with data available from at least two trials, and four comparisons were identified as below. In 2-dose-SP arms, women were given 2 doses of 1500 mg sulfadoxine and 75 mg pyrimethamine, at least 4 weeks apart, during pregnancy. While in monthly IPT-SP arms, women were usually enrolled into the trial during the second trimester and received the SP treatment monthly thereafter, with total 3 doses or more during pregnancy.

- 2-dose SP vs weekly chloroquine
- Monthly IPTp-SP vs 2-dose SP
- Monthly IPTp-DHA + PPQ vs monthly IPTp-SP
- Monthly IPTp-SP plus azithromycin vs similar regimen but without azithromycin

We used first-trimester weight as a proxy for maternal pre-pregnancy baseline weight to calculate weight gain during pregnancy. For women who did not have first-trimester weight, we developed several models to impute their first-trimester weight using weights measured later during pregnancy. The details of the model development, selection, and validation have been published elsewhere.^15^ Briefly, mixed-effects models and restricted cubic splines were used to impute weight at 9 weeks of gestation. We chose to impute weight at 9 weeks because it is consistent with the first available weight measure during pregnancy used in the INTERGROWTH-21st Study, an international research project that developed GWG standards among pre-pregnancy normal-weight women.^16^ Body mass index (BMI) was calculated by dividing pre-pregnancy (if available) or first-trimester weight (observed or imputed) in kilograms by the square of height in meters. For women aged ≥20 years old, we used the WHO BMI cutoffs to define underweight (BMI <18.5 kg/m^2^), normal weight (18.5 ≤ BMI <25.0 kg/m^2^), overweight (25.0 ≤ BMI <30.0 kg/m^2^), and obesity (BMI ≥30.0 kg/m^2^).^17^ For adolescent women (<20 years old), we used the WHO adolescent growth reference to define underweight (BMI-for-age Z-score: < −2), normal weight (BMI-for-age Z-score: −2 to < 1), overweight (BMI-for-age Z-score: 1–< 2), and obesity (BMI-for-age Z-score: ≥2).^18^

GWG at the time of last weight measure during pregnancy was calculated for each woman by subtracting pre-pregnancy or first-trimester weight from the last available weight measurement during pregnancy. Second, following the Institute of Medicine (IOM) 2009 recommendation,^19^ we estimated the expected weight gain for each woman at the time of their last observed weight measure using the following formula:

Recommended GWG = expected first-trimester weight gain/13.86∗(13.86—gestational age at first observed or imputed weight measurement) + [(gestational age at the last weight measurement—13.86 weeks) × recommended rate of GWG for the second and third trimester by BMI category based on IOM guidelines].

We assumed that the expected first-trimester weight gain was 2 kg for women with underweight and normal weight, 1 kg for women with overweight, and 0.5 kg for women with obesity.^20^ The recommended rates of GWG for the second and third trimesters were 0.51, 0.42, 0.28, and 0.22 kg per week for women with underweight, normal weight, overweight, and obesity, respectively.^19^ The percent adequacy of GWG was calculated by dividing the observed GWG at the time of the last weight measurement by the expected GWG for that week of gestation based on the IOM recommendations, multiplied by 100. This continuous outcome is independent of gestational age at the time of weight measure and has been employed previously.^20^

A weight measurement right before delivery was largely unavailable. The median time interval between last weight measurement during pregnancy and delivery was 3.1 (interquartile range: 1.1, 6.6) weeks. For each woman, we calculated her IOM-recommended GWG in grams at delivery based on her gestational age at delivery and BMI category. Then, we estimated total GWG at delivery in grams by multiplying the percent adequacy of GWG (estimated above) by IOM-recommended GWG in grams at delivery.

Analysis of variance were used to examine the associations of antimalarial intervention with GWG percent adequacy and total GWG in grams at delivery within each trial with antimalarial regimen as predictor. Patient characteristics by arm within each trial were summarised in Supplementary Table S2. Intention-to-treat strategy was used in the analysis. Mean differences (MDs) and 95% confidence intervals (CIs) obtained from the linear regression model were used to estimate the effect size across intervention arms. After analyses were completed for each trial, fixed-effect inverse-variance meta-analyses were conducted to pool results from individual trials with common intervention comparison together. Heterogeneity across trials was assessed using the *I*^*2*^ statistic, with thresholds of <25%, 50%, and >75% considered to represent low, moderate, and high heterogeneity, respectively.^21^ Forest plots were graphed to show the results from individual trials and the overall effect of meta-analysis.

Exploratory subgroup analyses by maternal pre-pregnancy BMI category (underweight, normal-weight, overweight or obesity) were conducted to examine whether the potential beneficial effect of IPTp-SP on GWG percent adequacy is dependent on maternal baseline nutritional status.

All individual trials have been approved by their respective ethics committees; detailed information on ethnical approval from each trial is shown in Supplementary Table S3. Two-tailed p-values <0.05 were considered significant. Statistical analyses were performed using SAS version 9.4 (SAS Institute) and Stata version 17.

Risk of bias for each trial was assessed using the Cochrane risk-of-bias tool, version 2 (RoB 2).^22^ Using this tool we examined five domains of bias including the randomization process, deviations from the intended intervention, missing outcome data, measurement of the outcome, and selection of reported results. RoB 2 Excel Macro Form was used to record the assessment results for each trial. Bias in each domain was summarised into “low risk”,” some concerns”, or “high risk”, and the summary figure was automatically created as output on the excel spreadsheet.

This systematic review has been registered in the International Prospective Register of Systematic Review: PROSPERO# CRD42023428794. Available from https://www.crd.york.ac.uk/prospero/display_record.php?ID=CRD42023428794. The study is reported as per the Preferred Reporting Items for Systematic Reviews and Meta-Analyses (PRISMA) guideline.

### Role of the funding source

The knowledge integration team at Gates Foundation supported the individual level data acquisition and harmonisation process. The funder of the study had no role in study design, data collection, data analysis, data interpretation, or writing of the report. EL and UP accessed and verified all data used in the study. All authors had final responsibility for the decision to submit for publication.

## Results

**Fig. 1:**
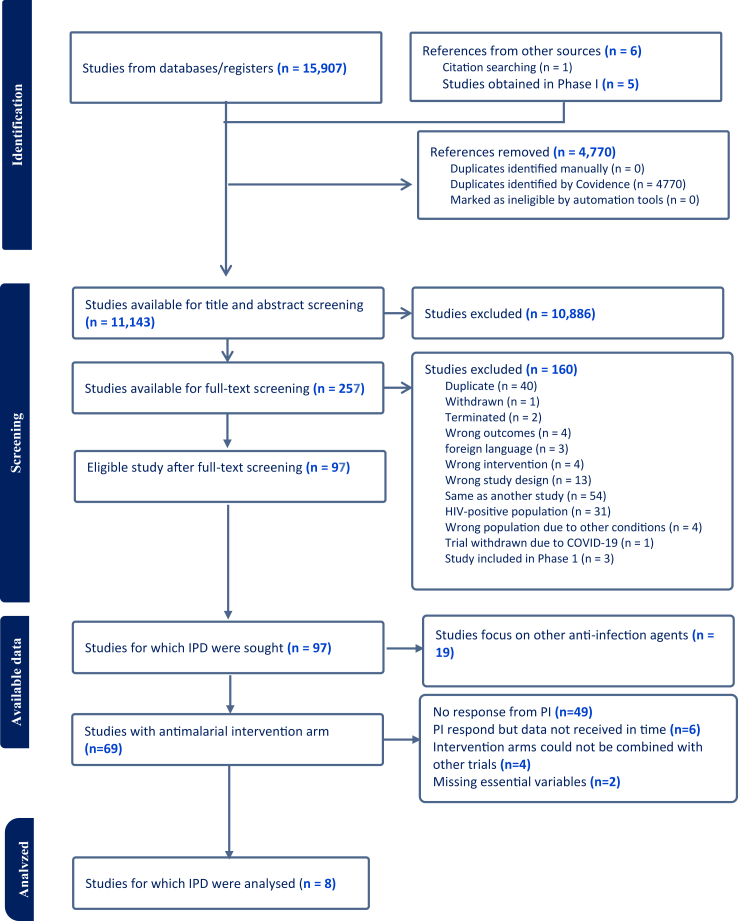
PRISMA flow diagram for individual participant data (IPD) meta-analysis.

We obtained IPD from 14 RCTs. IPD from 8 trials with 8550 pregnant woman participants were included in the meta-analysis (Fig. 1. PRISMA IPD flow diagram). The exclusion of the remaining 6 trials was primarily due to differences in intervention arms that couldn't be grouped with any other trials for meta-analysis or essential data such as maternal height or gestational age were missing, which prevented the imputation of GWG adequacy.

Seven of the 8 trials included in the current analysis were conducted in sub-Saharan Africa and one in Oceania. Two trials had 2-dose SP vs weekly chloroquine intervention arms,^23^^,^^24^ another two trials had monthly IPTp-SP vs 2-dose SP arms,^25^^,^^26^ and three trials had monthly IPTp-DHA + PPQ vs monthly IPTp-SP arms.^7^^,^^8^^,^^27^ Finally, two of the eight trials included interventions in combination with azithromycin vs without^28^^,^^29^ (Table 1).

**Table 1:** Characteristics of trials included in the meta-analysis of effects of antimalarial and antibiotics prophylaxis on GWG.

Author, year	Country	Study year	Weekly CQ	2-dose SP	IPTp-SP	IPTp-DHA + PPQ	IPTP-SP + AZ	#Patients	Weeks of gestation at enrollment, mean (SD)	BMI, kg/m^2^, mean (SD)
Roberfroid, 2008	Burkina Faso	2004–2006	X	X				1092	16.0 (6.2)	20.1 (2.0)
Valea, 2010	Burkina Faso	2006–2008		X	X			1110	15.8 (6.1)	20.2 (2.1)
Luntamo, 2010	Malawi	2004–2007		X	X		X	1280	20.2 (3.1)	20.6 (2.0)
Unger, 2015	Papua New Guinea	2010–2013	3-day SP + CQ				X	1908	21.4 (4.1)	21.1 (2.6)
Desai, 2015	Kenya	2012–2014			X	X		1233	21.6 (3.8)	21.3 (2.8)
Divala, 2018	Malawi	2012–2014	X	X				758	22.6 (2.8)	22.3 (2.8)
Kajubi, 2019	Uganda	2016–2017			X	X		662	15.3 (2.3)	21.8 (2.7)
Andronescu, 2021	Malawi	2017–2018			X	X		507	20.0 (3.1)	22.3 (3.4)

GWG, gestational weight gain; SP, Sulfadoxine-pyrimethamine; CQ, chloroquine; AZ, azithromycin; IPTp-SP, Intermittent preventive treatment with sulfadoxine-pyrimethamine; IPTp-DHA + PPQ, Intermittent preventive treatment with dihydroartemisinin-piperaquine; BMI, first trimester body mass index; SD, standard deviation.

**Fig. 2:**
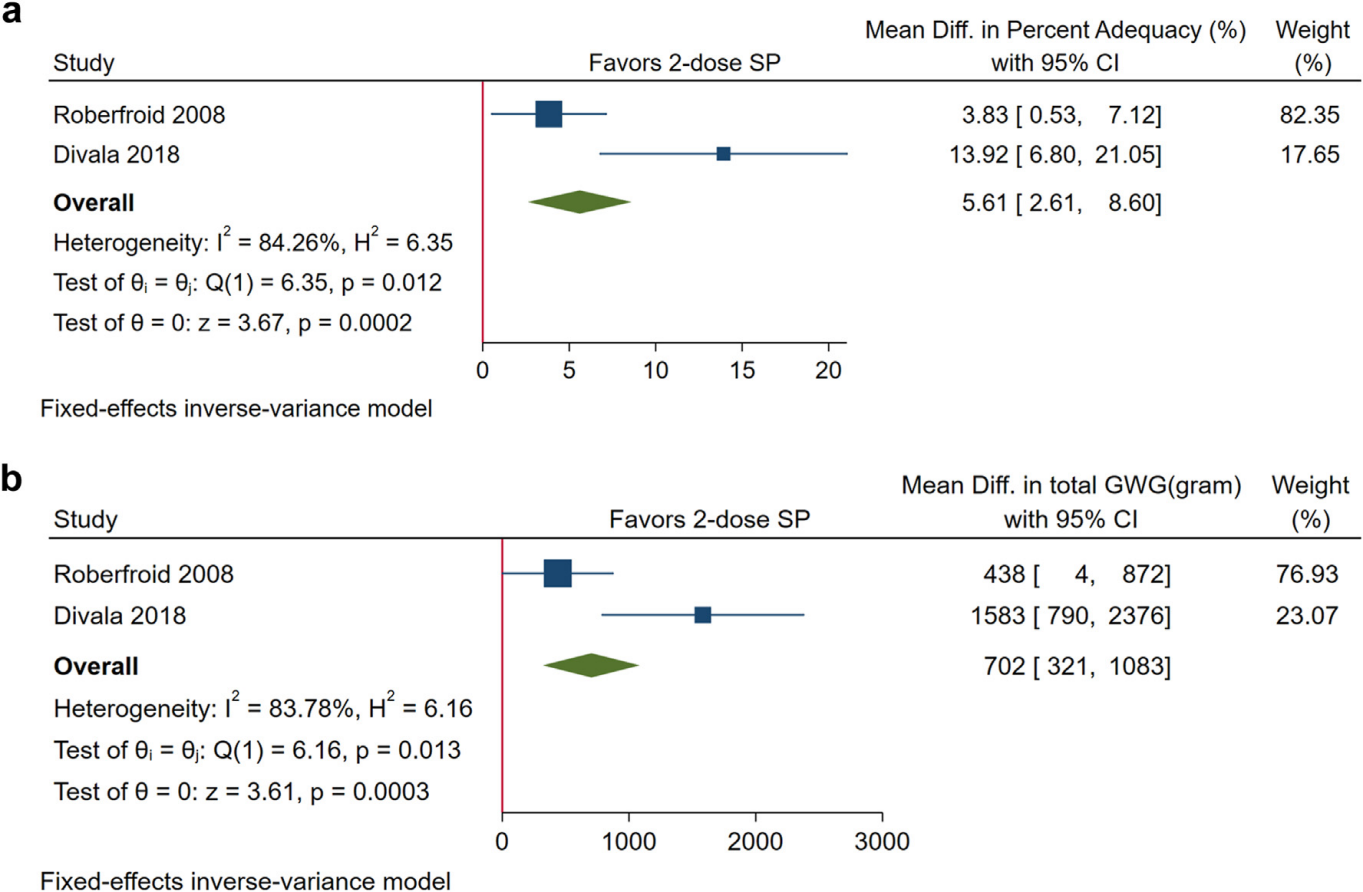
**a.** Forest plot of the effect of 2-dose sulfadoxine-pyrimethamine (SP) vs weekly chloroquine on gestational weight gain (GWG) percent adequacy shows that 2-dose SP is associated with a greater GWG percent adequacy. **b.** Forest plot of the effect of 2-dose sulfadoxine-pyrimethamine (SP) vs weekly chloroquine on total gestational weight gain (GWG) in grams at delivery shows that 2-dose SP is associated with an increased total GWG at delivery.

**Fig. 3:**
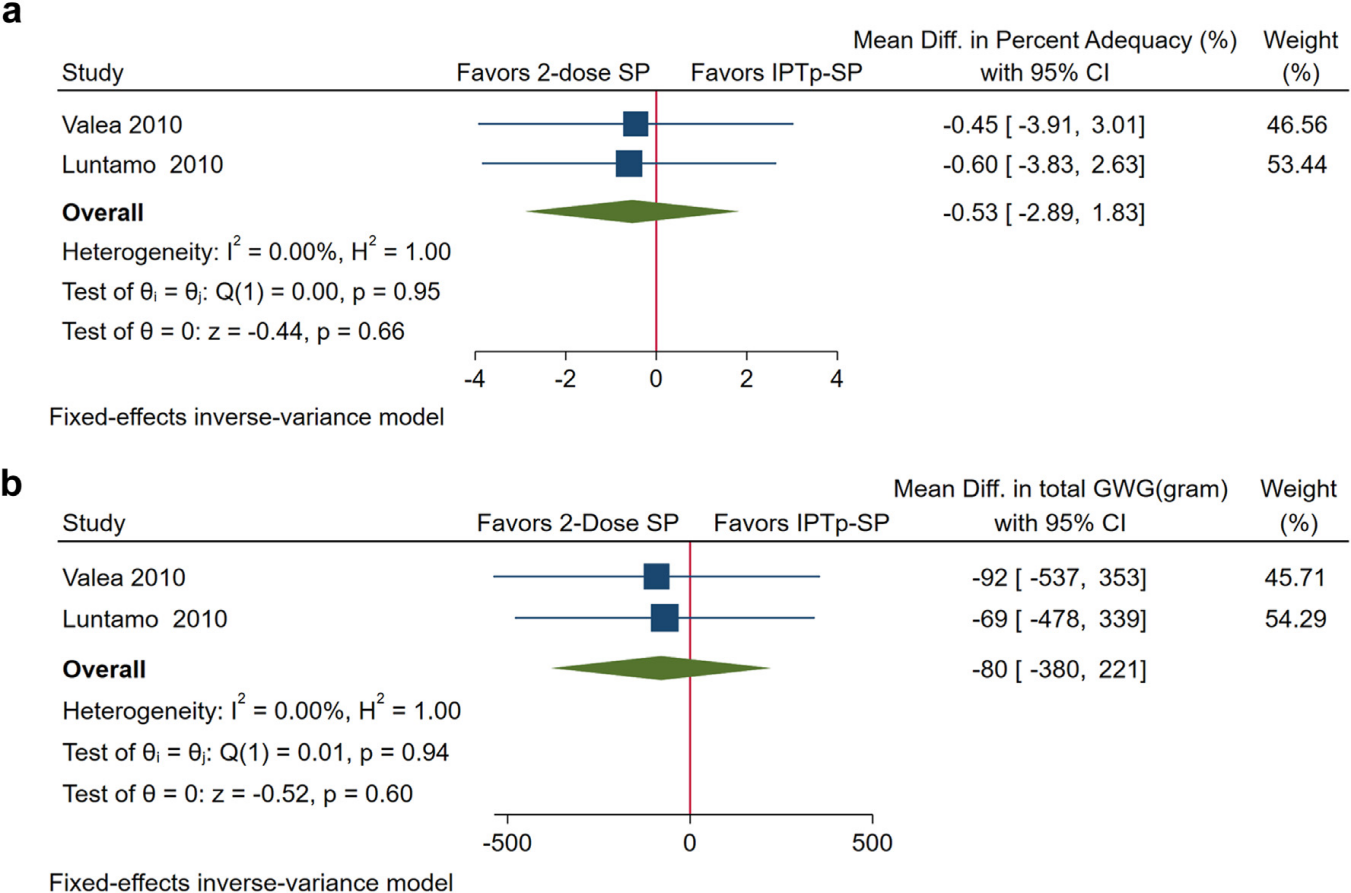
**a** Forest plot of the effect of monthly intermittent preventive treatment with sulfadoxine-pyrimethamine (IPTp-SP) vs 2-dose SP on gestational weight gain (GWG) percent adequacy shows that there is no difference between IPTp-SP and 2-dose SP. **b** Forest plot of the effect of monthly intermittent preventive treatment with sulfadoxine-pyrimethamine (IPTp-SP) vs 2-dose SP on total gestational weight gain (GWG) in grams at delivery shows that there is no difference between IPTp-SP and 2-dose SP.

Women who received 2-dose SP during pregnancy had a greater GWG percent adequacy (MD, 5.61%; 95% CI: 2.61%, 8.60%; P < 0.001) and total GWG at delivery (MD, 702 g; 95% CI: 321, 1083; P < 0.001) than those who received weekly chloroquine (Fig. 2a and b). We did not find a significant difference between monthly IPTp-SP and 2-dose SP in either GWG percent adequacy (MD, −0.53%; 95% CI: −2.89%, 1.83%; P = 0.66) or total GWG at delivery (MD, −80 g; 95% CI: −380,221; P = 0.60) (Fig. 3a and b).

**Fig. 4:**
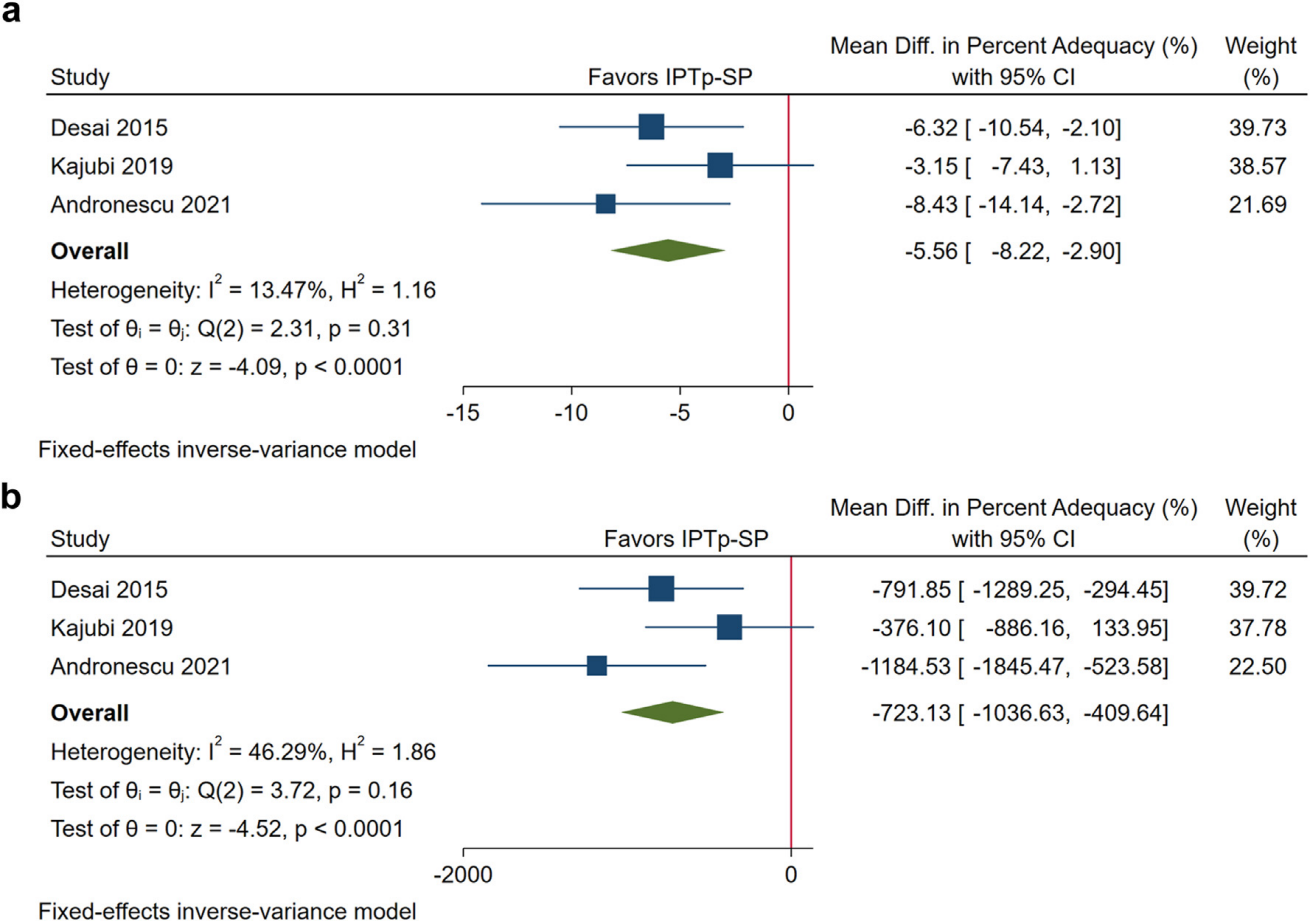
**a.** Forest plot of the effect of monthly intermittent preventive treatment with dihydroartemisinin piperaquine (IPTP-DHA + PPQ) vs monthly intermittent preventive treatment with sulfadoxine-pyrimethamine (IPTp-SP) on gestational weight gain (GWG) percent adequacy shows that DHA + PPQ is associated with a smaller GWG percent adequacy. **b.** Forest plot of the effect of monthly Intermittent Preventive Treatment with dihydroartemisinin piperaquine (IPTp-DHA + PPQ) vs monthly intermittent preventive treatment with sulfadoxine-pyrimethamine (IPTp-SP) on total gestational weight gain (GWG) in grams at delivery shows that DHA + PPQ is associated with a decreased total GWG at delivery.

Women who received monthly IPTp-DHA + PPQ had a significantly lower GWG percent adequacy (MD, −5.56%; 95% CI: −8.22%, −2.90%; P < 0.001) and total GWG (MD, −723 g; 95% CI: −1037, −410; P < 0.001) at delivery than those who received monthly IPTp-SP (Fig. 4a and b). To examine whether the adverse effect of IPTp-DHA + PPQ on GWG percent adequacy is dependent on maternal baseline nutritional status, subgroup analysis by BMI categories (underweight, normal weight, and overweight/obesity) were conducted. The results demonstrated that the MD (95% CI) was −8.91% (−15.34%, −2.48%) P = 0.0066, −5.05% (−7.71%, −2.35%) P = 0.0002, and −6.60% (−17.16%, 3.95%) P = 0.22 for underweight, normal weight, and overweight/obesity subgroups, respectively (Supplementary Figure S2). Meta-regression analysis showed that there is no significant difference across BMI categories with respect to the effect size (P = 0.44).

**Fig. 5:**
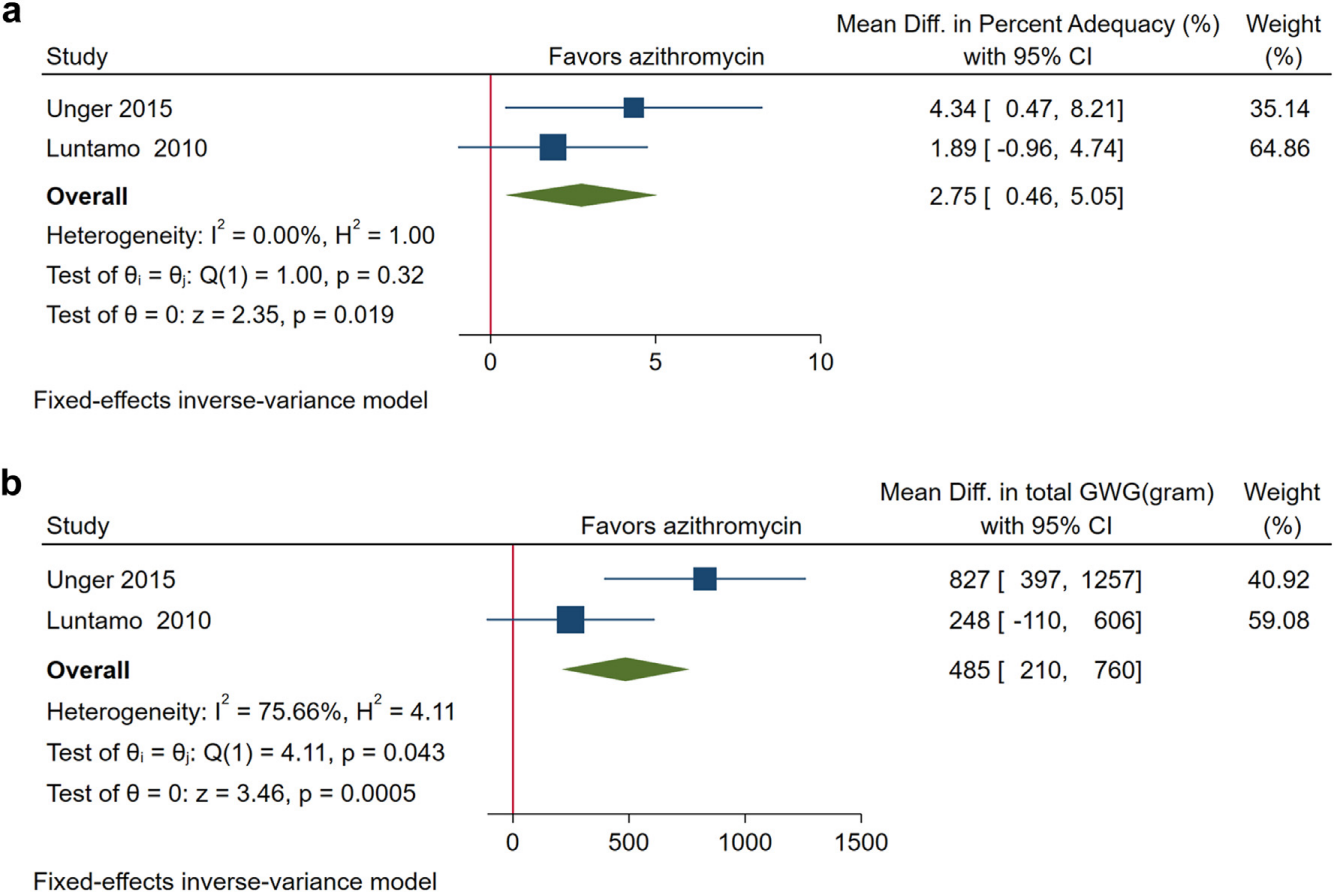
**a.** Forest plot of the effect of azithromycin on gestational weight gain (GWG) percent adequacy shows that adding azithromycin to an antimalarial regimen was associated with a greater GWG percent adequacy. **b.** Forest plot of the effect of azithromycin on total gestational weight gain (GWG) in grams at delivery shows that adding azithromycin to an antimalarial regimen was associated with an increased total GWG at delivery.

The inclusion of azithromycin in SP based regimen was associated with a significantly greater GWG percent adequacy (MD, 2.75%; 95% CI: 0.46%, 5.05%, P = 0.02) and total GWG at delivery (MD, 485 g; 95% CI: 210, 760; P < 0.001) compared to regimens without azithromycin (Fig. 5a and b).

Seven of the 8 trials included in the analysis were to have a low risk of bias, and one was assessed to have some concern of bias due to selection of the reported results (Supplementary Figure S1).

## Discussion

In this IPD meta-analysis, we found that the provision of 2-dose SP to pregnant women was associated with a significant increase in GWG compared to weekly chloroquine. There was no significant difference in GWG between 2-dose SP and monthly IPTp-SP. Furthermore, women who received monthly IPTp-SP in combination with azithromycin had a significantly greater GWG compared to those who received a similar antimalarial regimen but without azithromycin included. Notably, compared to monthly IPTp-SP, monthly IPTp-DHA + PPQ was associated with significantly lower GWG.

As an antimalarial medication, chloroquine has been widely used for the prevention and treatment of malaria. However, in the 1990s, its effectiveness was significantly compromised due to the rise of chloroquine resistance.^30^ This led to many health systems to completely remove chloroquine as an antimalarial medication.^31^ Over time, the unavailability of chloroquine gradually restored the susceptibility of malaria parasite to it. In the mid-2000s, there was evidence indicating that the sensitivity to chloroquine was once again increasing in sub-Saharan Africa.^32^^,^^33^ Since then, several trials have been conducted to evaluate the effectiveness of chloroquine compared to other antimalarial medications such as 2-dose or monthly SP. The results showed that monthly IPTp-SP was the most effective approach in preventing malaria and improving pregnancy outcomes.^24^^,^^34^^,^^35^ In our own study, we found that women who received 2-dose SP during pregnancy had a significantly higher gestational weight gain compared to those receiving weekly chloroquine. Despite the evidence of chloroquine regaining efficacy, it is not a suitable option for pregnant women due to the well-documented adverse events and poor compliance associated with its use.

We did not find a significant difference in GWG between 2-dose SP and monthly IPTp-SP. Due to the study design, women in the IPTp-SP arm only received a maximum of 3-doses of SP in one of the two trials included in the analysis.^25^ The limited number of SP doses the pregnant women received in the IPTp-SP arm likely reduced the power to detect the difference in GWG between IPTp-SP and 2-dose SP arms. Previous research has demonstrated that IPT with 3 or more doses of SP among pregnant women was associated with a higher birth weight and lower risk of low birthweight than the 2-dose SP regimen.^36^ Since 2013, the WHO has recommended that starting as early as possible in the second trimester, monthly IPTp-SP should be provided to all pregnant women in endemic areas to ensure that at least three doses are received during pregnancy.^6^

With the widespread adoption of monthly IPTp-SP in endemic regions, rising drug resistance has been a major challenge, especially in regions where two of the mutations associated with SP resistance, pfdhps540E and pfdhps581G, are prevalent.^37^ A recent systematic analysis of *P. falciparum* resistance to SP in Africa has demonstrated continued increase in SP resistance, particularly in eastern Africa.^38^ Despite this, IPTp-SP continues to maintain efficacy for prevention of low birthweight in most areas, with the exception of areas with the highest level of SP resistance.^39^ Furthermore, SP is not recommended for women who are in their first trimester due to possible teratogenic effects, and HIV-infected pregnant women on co-trimoxazole due to potential drug–drug interaction.^40^ Therefore, identifying new antimalarial medication in these circumstances is urgent. DHA + PPQ, an artemisinin-based combination therapy, has been evaluated as a promising alternative. Using IPD from existing trials, the current meta-analysis examined the effect of monthly IPTp-DHA + PPQ on GWG comparing to the standard monthly IPTp-SP, and our results show that monthly IPTp-DHA + PPQ was associated with significantly lower GWG compared to monthly IPTp-SP, consistent with previous negative findings on birth outcomes.^7^^,^^8^^,^^41^ Although a few trials from sub-Saharan Africa found that prevalence of malaria infection, placental malaria and clinical malaria were lower in monthly IPTp-DHA + PPQ than monthly IPTp-SP,^7^^,^^8^^,^^41^^,^^42^ the protective effect on malarial infection did not result in beneficial effect on birth outcomes. Trials from Kenya^7^ and Malawi^43^ even showed that IPTp-DHA + PPQ was associated with significantly lower birthweight and birthweight z score than IPTp-SP. A recent meta-analysis focused on comparing the two regimens further validated these findings^44^ It is thought that aside from its antimalarial properties, SP may also play a role in preventing and managing respiratory tract and sexual transmission infections and promoting maternal intestinal microbiome during pregnancy, which could potentially affect fetal growth.^13^ Furthermore, Waltmann et al.^43^ have demonstrated that the positive effect of IPTp-SP vs IPTp-DHA + PPQ on birthweight is mediated by GWG in the trial from Malawi. Our results on GWG are consistent with those previous findings on birthweight.

We found that including azithromycin in the antimalarial IPTp regimen was associated with significant increases in GWG. Our result is consistent with a previous finding from the Malawi trial that adding azithromycin to monthly IPTp-SP resulted in similar antimalarial effects,^11^ but it appeared to offer further benefits in reducing fetal and neonatal growth faltering.^45^ It is plausible that the improvement in fetal growth associated with azithromycin could be due to its antibiotic effects against other infections, such as respiratory tract infection, during pregnancy. In 2015, a Cochrane review concluded that antibiotic prophylaxis including azithromycin during the second and third trimester was effective in reducing risk of preterm delivery in pregnant women with previous preterm delivery or with bacterial vaginosis in the current pregnancy.^46^ However, a recent trial from Burkina Faso found that adding azithromycin to IPTp-SP did not offer further benefits in reducing low birthweight.^47^ It needs to be noted that azithromycin was not the only difference between the two comparison arms in one of the two trials included in the current analysis, thus the significant difference in GWG^10^ and birth outcomes^29^ observed in this trial conducted in Papua New Guinea could not be exclusively attributed to azithromycin since the two comparison arms were monthly IPTp-SP plus azithromycin vs one-dose SP plus chloroquine. Despite concerns that resistant organisms will develop during widespread use of antibiotics,^48^ the effect of azithromycin used as part of an IPTp regimen during pregnancy on maternal and child health outcomes in the areas of moderate to high *P. falciparum* transmission warrants further investigation.

The current study is the first meta-analysis to examine the effects of antimalarial IPTp on GWG using IPD obtained from existing RCTs in LMICs. Over the years, a variety of medications with different frequency and dose delivery strategies have been developed and evaluated to reduce potential adverse effects of malaria in pregnancy. To examine their effects on GWG, we sought to conduct a comprehensive review and summarise all possible comparisons between different IPTp and prophylaxis approaches. Although we were not able to obtain all the data we sought, our analyses included main comparisons among IPTp and prophylactic drugs commonly used during pregnancy.

Several limitations of this study need to be noted. First, the number of trials available for meta-analysis is limited, with most comparisons only including 2 trials. The lower number of trials significantly limits our ability to assess between-study heterogeneity and its source through meta-regression, as well as publication bias. Due to this limitation, we were likely underpowered to detect difference between compared medications/delivery strategies, especially for the comparison between monthly IPTp-SP and 2-dose SP. Second, during the updated literature in February 2025, we found one relevant trial which was published in 2024 and conducted in Nigeria. Pregnant women in this trial were randomised to receive either monthly IPTp-SP or two-dose SP during pregnancy. The results showed that monthly IPTp-SP is more effective in preventing malaria in pregnancy, including placental parasitemia. Given the uncertainty of the availability of weight and height measures and time-consuming process of obtaining individual level data, we were not able to include this trial in the current analysis.^49^ Third, all the trials included in our study, except for one, were conducted in sub-Saharan Africa, therefore our results may not be generalizable to pregnant women in other regions. Nevertheless, it is crucial to note that sub-Saharan Africa bears the highest burden of malaria-affected pregnancies. In fact, the IPTp is only recommended by WHO specifically for pregnant women this region. Hence, our findings hold significantly relevant within this context. Additionally, it is important to note that in all of these trials, the predominant species of malaria was *P. falciparum*, thus the potential benefit of IPTp or prophylaxis for other species like *P. vivax* remains uncertain.

In conclusion, our study found that IPTp-SP, either alone or in combination with azithromycin, was associated with a higher rate of gestational weight gain (GWG). These results align with previous findings on birth outcomes and support the WHO recommendation that pregnant women in malaria-endemic areas receive preventive treatment of monthly IPTp-SP starting from the second trimester. However, more efforts are needed to find alternative medications and strategies to decrease malaria infection and promote optimal GWG and birth outcomes in regions where *P. falciparum* resistance to SP is prevalent. More large-scale trials maybe considered to examine the effectiveness and safety of adding azithromycin or DHA + PPQ to IPTp-SP on malarial infection and birth outcome.

## Contributors

EJ and WWF conceptualised the study question. UP, SKN, VS, and BB facilitated data curation and harmonizations. EL conducted the statistical analysis and wrote the first draft of paper. EL, SS, DQ, MW, and WWF contributed to study methodology. DHH and WWF critically read and revised the paper. All other co-authors made the individual participant data available for the meta-analysis presented in this study and have read, commented, or edited the manuscript. All authors had full access to all the data in the study. EL and UP accessed and verified all data used in the study. All authors had final responsibility for the decision to submit for publication.

## Data sharing statement

Access to all individual-level data that comprise the pooled dataset use in this meta-analysis is restricted to approved individuals at Gates Foundation and the Harvard TH Chan School of Public Health based on the terms set forth in their Data Use agreement. Reasonable requests from qualified researchers will be considered for data sharing. Hese request should be submitted to ghp@hsph.harvard.edu.

## Declaration of interests

MD and JRG are on the DSMB for the APRIRE trial and the Pyrapreg trial, they co-chair the Roll Back Malaria, Malaria in Pregnancy Working group; SJR received grants from NHMRC, Australia, and NIH, USA; All other co-authors declare no competing interests.
