# Supplementary material for: Effect of intermittent preventive treatment during pregnancy with sulfadoxine-pyrimethamine on maternal gestational weight gain in low-income and middle-income countries: a systematic review and individual participant data meta-analysis of randomised clinical trials
Source: eClinicalMedicine. 2025 Jun 2;84:103279. doi: 10.1016/j.eclinm.2025.103279 (PMC12167835; doi:10.1016/j.eclinm.2025.103279)
Supplement: Supplemental Material [file mmc1.pdf]

Effect of intermittent preventive treatment during pregnancy with sulfadoxine-pyrimethamine on maternal gestational weight gain in low-income and middle-income countries: a systematic review and individual participant data meta-analysis of randomised clinical trials

Enju Liu et al

Online Supplementary Material

Literature search strategy

We systematically searched public health and interdisciplinary databases, such as PubMed, Embase, and Web of Science, to identify randomized controlled trials among pregnant women in low- and middle- income countries. There were no language restrictions.

Concept	PubMed Search terms
#1 Pregnancy	"Pregnancy"[Mesh] OR Pregnanc*[tiab] OR Pregnant[tiab] OR prenatal[tiab] OR gestation*[tiab] or antenatal[tiab] OR "Pregnant Women"[Mesh] OR gravid[tiab] OR obstetric[tiab] OR antepartum[tiab]
#2 Low- and middle-income countries	"Developing Countries"[MeSH] OR “developing countr*”[tiab] OR “developing nation*”[tiab] OR “less developed countr*”[tiab] OR “less developed nation*”[tiab] OR “third world nation*”[tiab] OR “third world countr*”[tiab] OR “under developed nation*”[tiab] OR “underdeveloped nation*”[tiab] OR “under developed countr*”[tiab] OR “underdeveloped countr*”[tiab] OR “middle income countr*”[tiab] OR “middle-income countr*”[tiab] OR “middle income nation*”[tiab] OR “middle-income nation*”[tiab] OR “low income countr*”[tiab] OR “low-income countr*”[tiab] OR “low income nation*”[tiab] OR “low-income nation*”[tiab] OR “poor countr*”[tiab] OR “poor nation*”[tiab] OR lmic[tiab] OR lmics[tiab] OR "Africa"[MeSH] OR "Asia"[MeSH] OR "South America"[MeSH] OR "Latin America"[MeSH] OR "Central America"[MeSH] OR africa[tiab] OR asia[tiab] OR “south america*”[tiab] OR “latin america*”[tiab] OR “central america*”[tiab] OR Afghanistan*[tiab] OR Albania*[tiab] OR Algeria*[tiab] OR Samoa*[tiab] OR Angola*[tiab] OR Armenia*[tiab] OR Azerbaijan*[tiab] OR Bangladesh*[tiab] OR Bengali[tiab] OR Belarus*[tiab] OR Belize[tiab] OR Benin[tiab] OR Bhutan*[tiab] OR Bolivia*[tiab] [list continues to include all World Bank Fiscal Year 2021 LMICs listed by name]
#3 Trials	"Clinical Trials as Topic"[Mesh] OR “Randomized Controlled Trial”[pt] OR Clinical Trial[pt] OR “Controlled Clinical Trial”[pt] OR "randomized controlled trials as topic"[MeSH] OR “controlled trial*”[tiab] OR intervention*[tiab] OR "random allocation"[MeSH] OR random*[tiab] OR trial*[tiab] OR "Clinical Trial Protocols as Topic"[Mesh] OR "Clinical Trial Protocol"[pt] OR "Clinical Study"[pt] OR "Clinical Studies as Topic"[Mesh] OR "Therapeutic Uses"[Mesh] OR "therapeutic use"[Subheading]
#4 Anti-infection agents	"Anti-Infective Agents"[Mesh] OR "Anti-Infective Agents"[Pharmacological Action] OR “anti-infective*”[tiab] OR “anti-infection”[tiab] OR "Anti-Bacterial Agents"[Mesh] OR "Anti-

	Bacterial Agents"[Pharmacological Action] OR “anti-bacterial*”[tiab] OR antibacterial*[tiab] OR "Antibiotic Prophylaxis"[Mesh] OR antibiotic*[tiab] OR "Azithromycin"[Mesh] OR azithromycin[tiab] OR "Antiviral Agents"[Mesh] OR "Antiviral Agents"[Pharmacological Action] OR antiviral*[tiab] OR "Anti-Retroviral Agents"[Mesh] OR "Anti-Retroviral Agents"[Pharmacological Action] OR “anti-retroviral*”[tiab] OR antiretroviral*[tiab] OR "Antiparasitic Agents"[Mesh] OR "Antiparasitic Agents"[Pharmacological Action] OR antiparasitic*[tiab] OR "Parasites"[Mesh] OR parasite*[tiab] OR deworm*[tiab] OR deworming[tiab] OR "Helminths"[Mesh] OR "Anthelmintics"[Mesh] OR "Anthelmintics"[Pharmacological Action] OR anthelmintic*[tiab] OR "Albendazole"[Mesh] OR Albendazole[tiab] OR "Antimalarials"[Mesh] OR "Antimalarials"[Pharmacological Action] OR antimalaria*[tiab] OR “anti-malaria*”[tiab] OR "Malaria"[Mesh] OR malaria*[tiab] OR "Mefloquine"[Mesh] OR Mefloquine[tiab] OR Lariam[tiab] OR "Sulfadoxine"[Mesh] OR Sulfadoxine[tiab] OR "Pyrimethamine"[Mesh] OR Pyrimethamine[tiab] OR Fansidar[tiab] OR "Chloroquine"[Mesh] OR chloroquine[tiab] OR "Antifungal Agents"[Mesh] OR "Antifungal Agents"[Pharmacological Action] OR “anti-fungal*”[tiab] OR antifungal*[tiab] OR "Vaccines"[Mesh] OR vaccine*[tiab] OR vaccinate*[tiab] OR vaccination*[tiab]
#5 Animal studies	Animals[Mesh] NOT (Animals[Mesh] AND Humans[Mesh])
Search strategy	#1 And #2 And #3 And #4 and Not #5

Supplementary figure 1. Summary of risk bias assessment

Supplementary figure 2. Subgroup analysis of the effect of IPTP-DHA+PPQ versus IPTp-SP on GWG percent adequacy, by BMI category. IPTp Intermittent Preventive Treatment during pregnancy; DHA+PPQ, dihydroartemisinin piperaquine; SP, sulfadoxine-pyrimethamine; GWG, gestational weight gain, BMI, body mass index.

Supplementary figure 1. Summary of risk bias assessment

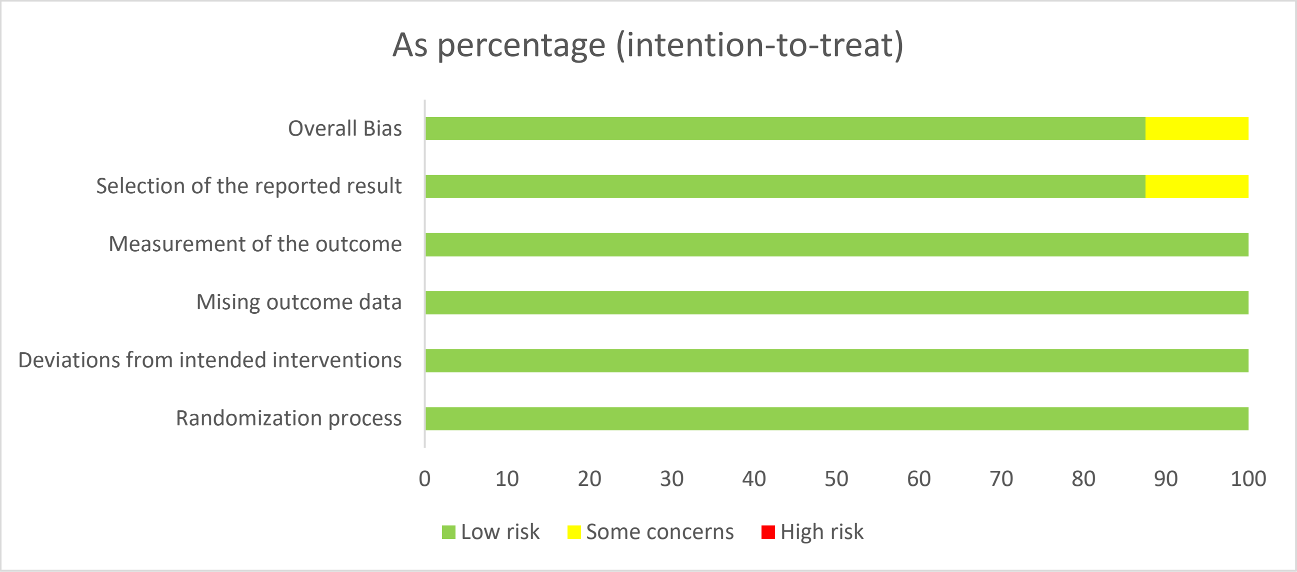

Supplementary figure 2. Subgroup analysis of the effect of IPTP-DHA+PPQ versus IPTp-SP on GWG percent adequacy, by BMI category. IPTp Intermittent Preventive Treatment during pregnancy; DHA+PPQ, dihydroartemisinin piperaquine; SP, sulfadoxine-pyrimethamine; GWG, gestational weight gain, BMI, body mass index.

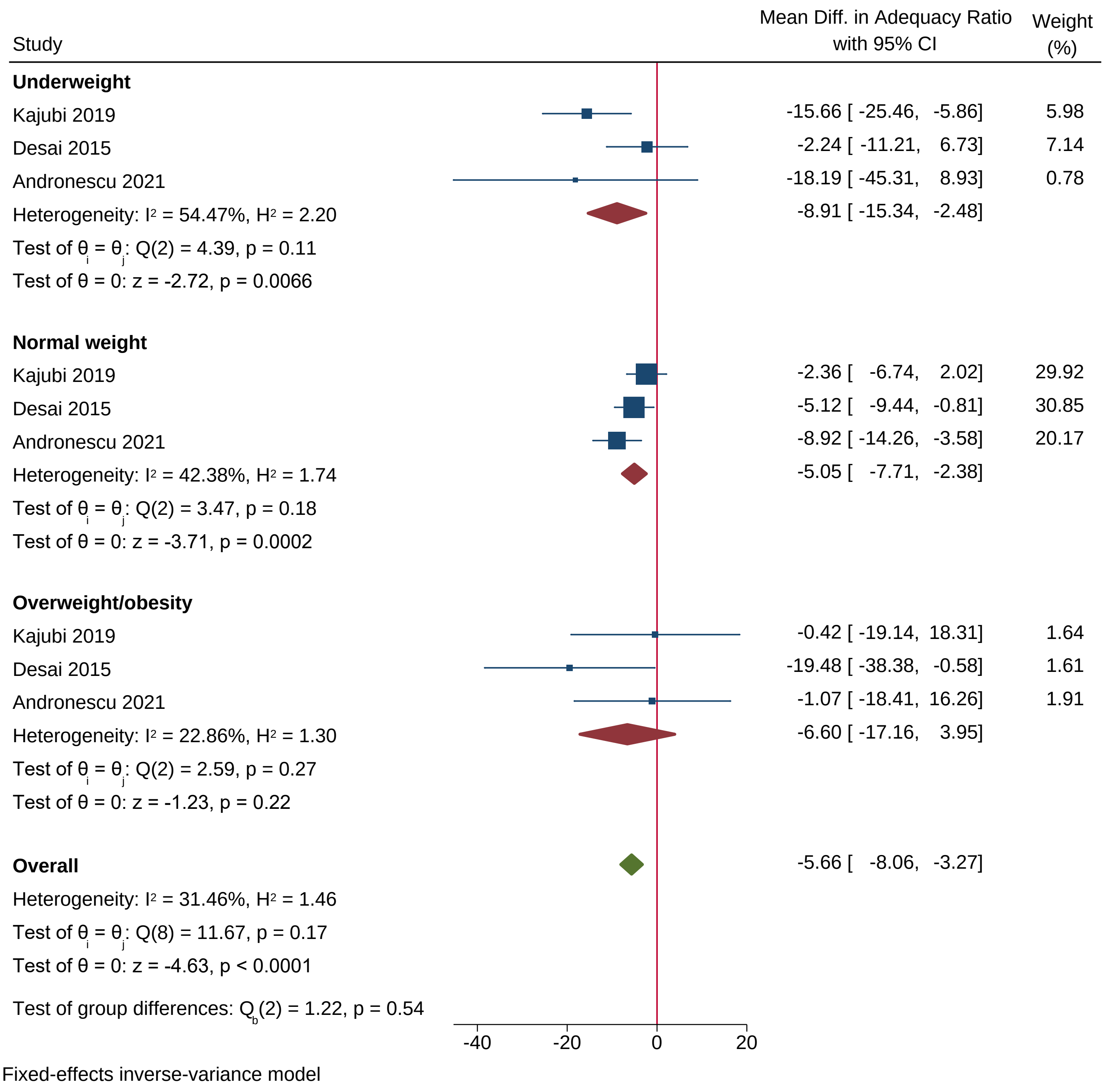

Supplementary table 1. Subject characteristics by trials (n=8550)

	Roberfroid 2008 (n=1092)	Valea 2010 (n=1110)	Luntamo 2010 (n=1280)	Unger 2015 (n=1908)	Desai 2015 (n=1233)	Divala TH 2018 (n=758)	Kajubi 2019 (n=662)	Andronescu 2021 (n=507)
Maternal age (years)	24.3 (6.2)	24.4 (6.3)	24.9 (6.5)	24.5 (5.4)	23.5 (5.7)	21.1 (3.4)	24.0 (5.9)	24.5 (6.2)
Maternal age								
- <20, %(n)	26.5 (287)	25.5 (283)	24.0 (307)	18.4 (352)	28.1 (347)	36.1 (274)	27.5 (182)	26.4 (134)
- 20 to 29, %(n)	51.9 (563)	53.5 (594)	52.3 (668)	63.4 (1209)	57.0 (703)	61.9 (469)	52.3 (346)	50.7 (257)
- 30+, %(n)	21.6 (234)	21.0 (233)	23.6 (302)	18.2 (347)	14.8 (183)	2.0 (15)	20.2 (134)	22.9 (116)
Gestational weeks at enrollment)	16.0 (6.2)	15.8 (6.1)	20.2 (3.1)	21.4 (4.1)	21.6 (3.8)	22.6 (2.8)	15.3 (2.3)	20.0 (3.1)
Gestational weeks at enrolment, median (IQR)	16.1 (10.4,21.3)	15.6 (10.7,20.9)	20.3 (18.0,22.6)	22.0 (18.9,24.6)	21.9 (19.1,24.6)	22.1 (21.0,24.0)	15.2 (13.3,17.1)	19.0 (17.0,22.0)
Gestational age at enrolment <20wks, %(n)	68.3 (746)	70.5 (783)	45.6 (584)	32.7 (623)	31.2 (385)	3.6 (27)	99.5 (659)	50.7 (257)
Gestational weeks at birth	39.0 (3.1)	38.9 (2.9)	38.6 (2.2)	38.8 (2.9)	39.1 (2.0)	38.9 (3.7)	39.0 (3.0)	38.4 (2.0)
BMI at first trimester	20.1 (2.0)	20.2 (2.1)	20.6 (2.0)	21.1 (2.6)	21.3 (2.8)	22.3 (2.8)	21.8 (2.7)	22.3 (3.4)
BMI at first trimester Median(IQR)	20.0 (18.8,21.4)	20.1 (18.7,21.5)	20.4 (19.2,21.9)	20.8 (19.4,22.5)	21.0 (19.4,22.9)	22.0 (20.3,23.8)	21.5 (19.9,23.1)	21.6 (20.1,23.6)
- underweight, %(n)	15.9 (174)	16.7 (185)	11.0 (141)	10.8 (207)	10.5 (129)	3.2 (24)	5.7 (38)	4.7 (24)
- normal, %(n)	82.9 (905)	80.8 (897)	86.8 (1111)	81.9 (1562)	80.3 (990)	82.5 (625)	82.3 (545)	78.9 (400)
- overweight, %(n)	1.1 (12)	2.4 (27)	2.1 (27)	7.0 (133)	8.3 (102)	12.9 (98)	11.0 (73)	13.8 (70)
- obese, %(n)	0.1 (1)	0.1 (1)	0.1 (1)	0.3 (6)	1.0 (12)	1.5 (11)	0.9 (6)	2.6 (13)
Maternal education <8yrs, %(n)	97.4 (1021)	97.8 (1064)		34.2 (615)	22.8 (280)		76.7 (508)	
Maternal height (cm)	162.1 (5.9)	162.6 (5.9)	155.0 (5.5)	154.2 (5.9)	164.2 (6.9)	157.3 (5.7)	158.6 (6.2)	157.4 (5.9)
Parity	2.6 (2.4)	2.6 (2.4)		2.2 (1.4)			2.1 (1.9)	

	Roberfroid 2008 (n=1092)	Valea 2010 (n=1110)	Luntamo 2010 (n=1280)	Unger 2015 (n=1908)	Desai 2015 (n=1233)	Divala TH 2018 (n=758)	Kajubi 2019 (n=662)	Andronescu 2021 (n=507)
Maternal HGB <11g/dL, %(n)	47.2 (486)	44.6 (492)	48.3 (618)	81.8 (1495)	59.1 (729)		32.9 (218)	54.9 (278)
Male infant, %(n)	50.2 (512)	49.8 (487)	51.6 (660)	44.4 (820)	50.6 (551)		49.5 (317)	54.3 (248)

Values are mean (SD) or median (Q1, Q3) for continuous variables; percentage (n) for categorical variables.

Values of polytomous variables may not sum to 100% due to rounding

Supplementary table 2 Subject characteristics by intervention arm within each trial (n=8550)

**Roberfroid 2008**	Weekly CQ (n=569)	2-dose SP (n=523)
Maternal age (years)	24.2 (6.1)	24.4 (6.3)
Maternal age		
- <20, %(n)	25.9 (146)	27.1 (141)
- 20 to 29, %(n)	52.8 (298)	51.0 (265)
- 30+, %(n)	21.3 (120)	21.9 (114)
Gestational weeks at enrollment	16.1 (6.3)	16.0 (6.1)
Gestational weeks at enrollment	16.7 (10.3,21.3)	16.0 (10.6,21.1)
Gestational age at enrolment <20wks, %(n)	68.2 (388)	68.5 (358)
Gestational weeks at birth	39.0 (2.9)	39.1 (3.3)
BMI at T1 or prepregnancy	20.0 (2.0)	20.3 (2.0)
BMI at T1 or prepregnancy	19.9 (18.6,21.3)	20.1 (18.9,21.6)
BMI at T1 or prepregnancy,categorical		
- underweight, %(n)	18.1 (103)	13.6 (71)
- normal, %(n)	81.0 (461)	84.9 (444)
- overweight, %(n)	0.9 (5)	1.3 (7)
- obese, %(n)	0.0 (0)	0.2 (1)
Maternal education (years)	0.9 (2.6)	1.0 (2.8)
Maternal education (years)	0.0 (0.0,0.0)	0.0 (0.0,0.0)
Maternal education <8yrs, %(n)	97.8 (532)	97.0 (489)
Maternal height (cm)	162.0 (5.7)	162.2 (6.0)
Maternal height <150cm, %(n)	0.9 (5)	1.3 (7)
Parity	2.6 (2.4)	2.6 (2.4)
Parity	2.0 (1.0,4.0)	2.0 (1.0,4.0)
Parity, categorical		
- 0, %(n)	21.8 (124)	20.3 (106)
- 1, %(n)	19.2 (109)	20.3 (106)
- 2+, %(n)	59.1 (336)	59.5 (311)
Maternal hemoglobin (g/dL)	11.1 (1.7)	11.1 (1.7)
Maternal HGB <11g/dL, %(n)	49.7 (264)	44.6 (222)
Compliance (%)	76.7 (19.7)	79.0 (18.0)
Adherence <90%, %(n)	70.5 (389)	69.2 (355)
Male infant, %(n)	48.4 (253)	52.1 (259)
GWG percent adequacy,%	57.1 (26.6)	60.9 (29.1)
GWG at delivery in gram	7340.8 (3448.9)	7778.7 (3814.8)

Values are mean (SD) or median (Q1, Q3) for continuous variables; percentage (n) for categorical variables.

Values of polytomous variables may not sum to 100% due to rounding

**Valea 2010**	2-dose SP(n=548)	IPTp-SP (n=562)
Maternal age (years)	24.7 (6.5)	24.2 (6.1)
Maternal age		
- <20, %(n)	25.4 (139)	25.6 (144)
- 20 to 29, %(n)	51.8 (284)	55.2 (310)
- 30+, %(n)	22.8 (125)	19.2 (108)
Gestational weeks at enrollment	15.9 (6.0)	15.6 (6.1)
Gestational weeks at enrollment	15.8 (10.7,20.9)	15.3 (9.9,20.9)
Gestational age at enrolment <20wks, %(n)	71.0 (389)	70.1 (394)
Gestational weeks at birth	38.9 (2.8)	39.0 (3.0)
BMI at T1 or prepregnancy	20.2 (2.2)	20.2 (2.0)
BMI at T1 or prepregnancy	20.1 (18.6,21.6)	20.1 (18.8,21.5)
BMI at T1 or prepregnancy,categorical		
- underweight, %(n)	17.9 (98)	15.5 (87)
- normal, %(n)	79.4 (435)	82.2 (462)
- overweight, %(n)	2.6 (14)	2.3 (13)
- obese, %(n)	0.2 (1)	0.0 (0)
Maternal education (years)	1.0 (2.7)	0.7 (2.3)
Maternal education (years)	0.0 (0.0,0.0)	0.0 (0.0,0.0)
Maternal education <8yrs, %(n)	97.6 (523)	98.0 (541)
Maternal height (cm)	162.5 (5.9)	162.7 (5.9)
Maternal height <150cm, %(n)	1.5 (8)	1.2 (7)
Parity	2.7 (2.5)	2.6 (2.3)
Parity	2.0 (1.0,4.0)	2.0 (1.0,4.0)
Parity, categorical		
- 0, %(n)	21.0 (115)	19.9 (112)
- 1, %(n)	19.3 (106)	23.3 (131)
- 2+, %(n)	59.7 (327)	56.8 (319)
Maternal hemoglobin (g/dL)	11.1 (1.5)	11.1 (1.6)
Maternal HGB <11g/dL, %(n)	44.6 (244)	44.6 (248)
Compliance (%)	79.2 (18.2)	79.7 (17.1)
Adherence <90%, %(n)	67.8 (367)	67.9 (376)
Male infant, %(n)	49.0 (240)	50.7 (247)
GWG percent adequacy, %	59.2 (29.1)	58.8 (29.7)

**Valea 2010**	2-dose SP(n=548)	IPTp-SP (n=562)
GWG at delivery in gram	7514.7 (3734.6)	7422.9 (3788.0)

Values are mean (SD) or median (Q1, Q3) for continuous variables; percentage (n) for categorical variables.

Values of polytomous variables may not sum to 100% due to rounding

**Luntamo 2010**	2-dose SP (n=421)	IPTp-SP (n=430)	IPTP-SP + AZ (n=429)
Maternal age (years)	24.8 (6.8)	25.0 (6.7)	24.8 (5.9)
Maternal age			
- <20, %(n)	27.9 (117)	25.2 (108)	19.2 (82)
- 20 to 29, %(n)	47.9 (201)	49.7 (213)	59.3 (254)
- 30+, %(n)	24.3 (102)	25.2 (108)	21.5 (92)
Gestational weeks at enrollment	20.4 (3.0)	20.2 (3.2)	20.1 (3.1)
Gestational weeks at enrollment	20.4 (18.1,22.7)	20.1 (17.9,22.6)	20.1 (17.9,22.4)
Gestational age at enrolment <20wks, %(n)	42.5 (179)	46.5 (200)	47.8 (205)
Gestational weeks at birth	38.4 (2.1)	38.5 (2.4)	38.8 (2.1)
BMI at T1 or prepregnancy	20.4 (2.0)	20.6 (2.1)	20.7 (2.1)
BMI at T1 or prepregnancy	20.2 (19.1,21.6)	20.5 (19.2,21.8)	20.6 (19.4,22.1)
BMI at T1 or prepregnancy,categorical			
- underweight, %(n)	11.6 (49)	11.2 (48)	10.3 (44)
- normal, %(n)	86.5 (364)	86.5 (372)	87.4 (375)
- overweight, %(n)	1.9 (8)	2.3 (10)	2.1 (9)
- obese, %(n)	0.0 (0)	0.0 (0)	0.2 (1)
Maternal height (cm)	155.0 (5.6)	154.8 (5.5)	155.3 (5.6)
Maternal height <150cm, %(n)	16.6 (70)	18.6 (80)	16.3 (70)
Maternal hemoglobin (g/dL)	11.0 (1.9)	11.1 (1.7)	10.9 (2.0)
Maternal HGB <11g/dL, %(n)	48.9 (206)	47.0 (202)	49.0 (210)
Male infant, %(n)	50.7 (213)	54.7 (235)	49.4 (212)
GWG percent adequacy, %	68.3 (24.2)	67.7 (23.9)	70.9 (25.6)
GWG at delivery in gram	8508.8 (3076.7)	8439.4 (2998.2)	8863.3 (3176.0)

Values are mean (SD) or median (Q1, Q3) for continuous variables; percentage (n) for categorical variables.

Values of polytomous variables may not sum to 100% due to rounding

**Unger 2015**	3-day SP+CQ (n=944)	IPTP-SP + AZ (n=964)
Maternal age (years)	24.7 (5.5)	24.3 (5.4)

**Unger 2015**	3-day SP+CQ (n=944)	IPTP-SP + AZ (n=964)
Maternal age		
- <20, %(n)	17.5 (165)	19.4 (187)
- 20 to 29, %(n)	63.7 (601)	63.1 (608)
- 30+, %(n)	18.9 (178)	17.5 (169)
Gestational weeks at enrollment	21.4 (4.1)	21.5 (4.1)
Gestational weeks at enrollment	21.9 (19.0,24.4)	22.1 (18.9,24.9)
Gestational age at enrolment <20wks, %(n)	31.9 (301)	33.4 (322)
Gestational weeks at birth	38.6 (3.1)	39.0 (2.7)
BMI at T1 or prepregnancy	21.1 (2.6)	21.1 (2.5)
BMI at T1 or prepregnancy	20.8 (19.4,22.6)	20.7 (19.3,22.5)
BMI at T1 or prepregnancy,categorical		
- underweight, %(n)	10.6 (100)	11.1 (107)
- normal, %(n)	82.0 (774)	81.7 (788)
- overweight, %(n)	7.0 (66)	7.0 (67)
- obese, %(n)	0.4 (4)	0.2 (2)
Maternal education (years)	7.9 (2.7)	8.1 (2.6)
Maternal education (years)	8.0 (6.0,10.0)	8.0 (6.0,10.0)
Maternal education <8yrs, %(n)	35.9 (319)	32.5 (296)
Maternal height (cm)	154.4 (6.1)	153.9 (5.6)
Maternal height <150cm, %(n)	18.5 (175)	19.8 (191)
Parity	2.2 (1.4)	2.2 (1.4)
Parity	2.0 (1.0,3.0)	2.0 (1.0,3.0)
Parity, categorical		
- 1, %(n)	40.9 (186)	40.7 (180)
- 2+, %(n)	59.1 (269)	59.3 (262)
Maternal hemoglobin (g/dL)	9.7 (1.5)	9.7 (1.4)
Maternal HGB <11g/dL, %(n)	80.5 (727)	83.1 (768)
Compliance (%)	93.0 (16.5)	93.2 (16.2)
Adherence <90%, %(n)	17.4 (164)	16.9 (163)
Male infant, %(n)	43.3 (394)	45.5 (426)
GWG percent adequacy,%	100.4 (44.0)	104.7 (42.4)
GWG at delivery in gram	11998.0 (4797.1)	12825.1 (4790.0)

Values are mean (SD) or median (Q1, Q3) for continuous variables; percentage (n) for categorical variables.

Values of polytomous variables may not sum to 100% due to rounding

**Desai 2015**	IPTp-SP (n=417)	IPTp-DP (n=404)
Maternal age (years)	23.6 (5.9)	23.6 (5.4)
Maternal age		
- <20, %(n)	28.1 (117)	27.5 (111)
- 20 to 29, %(n)	56.4 (235)	58.4 (236)
- 30+, %(n)	15.6 (65)	14.1 (57)
Gestational weeks at enrollment	21.5 (4.0)	21.6 (3.6)
Gestational weeks at enrollment	21.7 (19.0,24.7)	21.9 (19.1,24.6)
Gestational age at enrolment <20wks, %(n)	32.4 (135)	31.2 (126)
Gestational weeks at birth	39.1 (1.8)	39.0 (2.3)
BMI at T1 or prepregnancy	21.4 (3.1)	21.3 (2.8)
BMI at T1 or prepregnancy	21.0 (19.3,23.1)	20.9 (19.4,22.8)
BMI at T1 or prepregnancy,categorical		
- underweight, %(n)	12.0 (50)	9.2 (37)
- normal, %(n)	77.7 (324)	81.7 (330)
- overweight, %(n)	9.1 (38)	8.4 (34)
- obese, %(n)	1.2 (5)	0.7 (3)
Maternal education (years)	9.0 (2.4)	9.0 (2.5)
Maternal education (years)	8.0 (8.0,11.0)	8.0 (8.0,11.0)
Maternal height (cm)	164.2 (6.9)	164.6 (6.7)
Maternal height <150cm, %(n)	1.4 (6)	1.2 (5)
Maternal hemoglobin (g/dL)	10.6 (1.5)	10.6 (1.5)
Maternal HGB <11g/dL, %(n)	59.0 (246)	56.9 (230)
Male infant, %(n)	50.5 (187)	50.3 (180)
GWG percent adequacy, %	80.4 (33.3)	74.1 (28.1)
GWG at delivery in gram	9664.0 (3679.7)	8872.2 (3175.9)

Values are mean (SD) or median (Q1, Q3) for continuous variables; percentage (n) for categorical variables.

Values of polytomous variables may not sum to 100% due to rounding

**Divala 2018**	Weekly CQ (n=252)	2-dose SP (n=248)
Maternal age (years)	20.9 (3.6)	21.1 (3.2)
Maternal age		
- <20, %(n)	40.9 (103)	35.9 (89)
- 20 to 29, %(n)	56.0 (141)	62.9 (156)

**Divala 2018**	Weekly CQ (n=252)	2-dose SP (n=248)
- 30+, %(n)	3.2 (8)	1.2 (3)
Gestational weeks at enrollment	22.8 (2.6)	22.4 (2.9)
Gestational weeks at enrollment	22.3 (21.3,24.0)	21.9 (20.9,23.4)
Gestational age at enrolment <20wks, %(n)	4.4 (11)	2.4 (6)
Gestational weeks at birth	38.7 (3.2)	39.1 (3.7)
BMI at T1 or prepregnancy	22.3 (2.5)	22.1 (2.8)
BMI at T1 or prepregnancy	22.3 (20.5,23.9)	21.8 (20.2,23.5)
BMI at T1 or prepregnancy,categorical		
- underweight, %(n)	2.4 (6)	3.6 (9)
- normal, %(n)	82.9 (209)	82.3 (204)
- overweight, %(n)	14.3 (36)	12.1 (30)
- obese, %(n)	0.4 (1)	2.0 (5)
Maternal height (cm)	157.1 (5.4)	157.5 (5.8)
Maternal height <150cm, %(n)	7.9 (20)	8.5 (21)
GWG percent adequacy, %	68.3 (40.6)	82.2 (40.7)
GWG at delivery in gram	7982.3 (4573.5)	9565.3 (4470.8)

Values are mean (SD) or median (Q1, Q3) for continuous variables; percentage (n) for categorical variables.

Values of polytomous variables may not sum to 100% due to rounding

**Kajubi 2019**	IPTp-SP (n=329)	IPTp-DP (n=333)
Maternal age (years)	24.0 (6.0)	23.9 (5.8)
Maternal age		
- <20, %(n)	28.0 (92)	27.0 (90)
- 20 to 29, %(n)	52.6 (173)	52.0 (173)
- 30+, %(n)	19.5 (64)	21.0 (70)
Gestational weeks at enrollment	15.4 (2.3)	15.2 (2.3)
Gestational weeks at enrollment	15.4 (13.3,17.4)	15.0 (13.1,16.9)
Gestational age at enrolment <20wks, %(n)	99.1 (326)	100.0 (333)
Gestational weeks at birth	39.1 (2.7)	38.9 (3.2)
BMI at T1 or prepregnancy	21.8 (3.0)	21.7 (2.5)
BMI at T1 or prepregnancy	21.4 (19.8,23.0)	21.5 (20.0,23.1)
BMI at T1 or prepregnancy,categorical		
- underweight, %(n)	5.8 (19)	5.7 (19)
- normal, %(n)	80.5 (265)	84.1 (280)

Kajubi 2019	IPTp-SP (n=329)	IPTp-DP (n=333)
- overweight, %(n)	12.2 (40)	9.9 (33)
- obese, %(n)	1.5 (5)	0.3 (1)
Maternal education (years)	7.3 (3.3)	7.7 (3.0)
Maternal education (years)	7.0 (7.0,7.0)	7.0 (7.0,7.0)
Maternal education <8yrs, %(n)	76.9 (253)	76.6 (255)
Maternal height (cm)	158.7 (6.4)	158.6 (6.0)
Maternal height <150cm, %(n)	7.0 (23)	6.0 (20)
Parity	2.1 (1.9)	2.1 (1.9)
Parity	2.0 (0.0,3.0)	1.0 (1.0,3.0)
Parity, categorical		
- 0, %(n)	26.7 (88)	24.6 (82)
- 1, %(n)	20.7 (68)	25.8 (86)
- 2+, %(n)	52.6 (173)	49.5 (165)
Maternal hemoglobin (g/dL)	11.4 (1.4)	11.4 (1.2)
Maternal HGB <11g/dL, %(n)	33.4 (110)	32.4 (108)
Male infant, %(n)	52.1 (165)	47.1 (152)
GWG percent adequacy, %	61.9 (27.6)	58.8 (28.6)
GWG at delivery in gram	7434.4 (3262.0)	7058.3 (3430.5)

Values are mean (SD) or median (Q1, Q3) for continuous variables; percentage (n) for categorical variables.

Values of polytomous variables may not sum to 100% due to rounding

Andronescu 2021	IPTp-SP (n=255)	IPTp-DP (n=252)
Maternal age (years)	24.3 (6.3)	24.7 (6.2)
Maternal age		
- <20, %(n)	29.0 (74)	23.8 (60)
- 20 to 29, %(n)	47.8 (122)	53.6 (135)
- 30+, %(n)	23.1 (59)	22.6 (57)
Gestational weeks at enrollment	20.0 (3.2)	20.0 (3.1)
Gestational weeks at enrollment	19.0 (17.0,22.0)	20.0 (18.0,22.0)
Gestational age at enrolment <20wks, %(n)	51.8 (132)	49.6 (125)
Gestational weeks at birth	38.6 (1.9)	38.1 (2.0)
BMI at T1 or prepregnancy	22.4 (3.2)	22.3 (3.5)
BMI at T1 or prepregnancy	21.8 (20.3,23.7)	21.6 (20.0,23.6)

**Andronescu 2021**	IPTp-SP (n=255)	IPTp-DP (n=252)
BMI at T1 or prepregnancy,categorical		
- underweight, %(n)	3.9 (10)	5.6 (14)
- normal, %(n)	79.2 (202)	78.6 (198)
- overweight, %(n)	14.5 (37)	13.1 (33)
- obese, %(n)	2.4 (6)	2.8 (7)
Maternal height (cm)	157.3 (6.2)	157.5 (5.7)
Maternal height <150cm, %(n)	4.3 (11)	6.3 (16)
Maternal hemoglobin (g/dL)	10.8 (1.4)	10.6 (1.3)
Maternal HGB <11g/dL, %(n)	54.1 (138)	55.8 (140)
Male infant, %(n)	56.0 (130)	52.4 (118)
GWG percent adequacy, %	89.6 (33.4)	81.2 (32.2)
GWG at delivery in gram	10180.9 (3839.9)	8996.4 (3358.8)

Values are mean (SD) or median (Q1, Q3) for continuous variables; percentage (n) for categorical variables.

Values of polytomous variables may not sum to 100% due to rounding

Supplementary table 3. Ethical approval information by trial.

Author, year	Country	Study year	Human subject IRB approval information
Roberfroid 2008	Burkina Faso	2004-2006	The study was approved by the ethics committees of the Center Muraz, Bobo-Dioulasso, Burkina Faso, and the Institute of Tropical Medicine, Antwerp, Belgium (Ref 02 35 4 399). The study purpose and procedures were explained in the local language: Bwamu, Moré, or Dioula, and a signed informed consent was sought.
Valea 2010	Burkina Faso	2006-2008	The protocol was approved by the Ethical Committees of the Centre Muraz, Bobo-Dioulasso, Burkina Faso, and the Institute of Tropical Medicine, Antwerp, Belgium. The study was registered at http://ClinicalTrial.gov registry (identifier: NCT00909974). Written informed consent was obtained from those agreeing to participate to the study after explaining the study purpose and procedures
Luntamo 2010	Malawi	2004-2007	The protocol was approved by the College of Medicine Research and Ethics Committee, Malawi (original trial on Apr 30, 2003 and the follow-up on Feb 22, 2006) and the Ethical Committee of Pirkanmaa Hospital District, Finland (original trial on Apr 29, 2003 and the follow up on May 9, 2006). The trial was registered with the U.S. National Library of Medicine (http://www.clinicaltrials.gov) with trial identification NCT00131235. Only participants who signed or thumb-printed an informed consent form were enrolled in the study.
Unger 2015	Papua New Guinea	2010-2013	Ethical approval was obtained from the PNG Institute of Medical Research Institutional Review Board (0815), the PNG Medical Research Advisory Council (08.01) and the Melbourne Health Human Research Ethics Committee (2008.162). The clinical trial was registered with the U.S. National Institutes of Health Clinical Trials Registry (Clinicaltrials.gov, registration NCT01136850). All participants have signed the informed consent form.
Desai 2015	Kenya	2012-2014	The study protocol received ethics approval from the Kenya Medical Research Institute (2179) and the US Centers for Disease Control and Prevention (6207). This study is registered with ClinicalTrials.gov, number NCT01669941. All study participants signed the consent form
Divala 2018	Malawi	2012-2014	The Institutional Review Board at the University of Maryland, the Research and Ethics Committee at the University of Malawi College of Medicine, and the Malawi Pharmacy Medicines and Poisons Board reviewed and approved the study protocol and informed consent forms. We obtained written

Author, year	Country	Study year	Human subject IRB approval information
			informed consent from every participant before undertaking any study procedures. This trial is registered with ClinicalTrials.gov, number NCT01443130.
Kajubi 2019	Uganda	2016-2017	The study was approved by the ethics committees of Makerere University School of Biomedical Sciences (Kampala, Uganda), review number SBS-342, the Uganda National Council for Science and Technology (Kampala, Uganda), review number HS-2052, and the University of California San Francisco (San Francisco, CA, USA), number 16-18679. All study participants provided written informed consent.
Andronescu, 2021	Malawi	2017-2018	The study was approved by the University of Malawi College of Medicine Research and Ethics Committee (COMREC; Blantyre, Malawi, P.02/16/1872) and the Centers for Disease Control and Prevention institutional review board (Atlanta, GA, 6836). Written informed consent was obtained from all participating women. Clinical Trials Registration. NCT03009526
